# CD19 CAR-T Cell Therapy Induced Immunotherapy Associated Interstitial Pneumonitis: A Case Report

**DOI:** 10.3389/fimmu.2022.778192

**Published:** 2022-01-27

**Authors:** Zhuanyi Sun, Caiqin Xie, Hui Liu, Xianggui Yuan

**Affiliations:** Department of Hematology, The Second Affiliated Hospital, College of Medicine, Zhejiang University, Hangzhou, China

**Keywords:** immunotherapy-associated pneumonitis, interstitial pneumonitis, chimeric antigen receptor-modified T cell, diffuse large B-cell lymphoma, intravenous immunoglobulin

## Abstract

**Background:**

Chimeric antigen receptor-modified T cells (CAR-T) targeting CD19 has produced a high durable response in refractory or relapsed diffuse large B-cell lymphoma. Besides well-known cytokine release syndrome (CRS) or immune effector cell–associated neurotoxicity syndrome during CAR-T cell therapy, there were several rarely encountered fatal complications.

**Case Report:**

A 63-year-old male patient with refractory EBV-positive diffuse large B-cell lymphoma, developed interstitial pneumonitis with prolonged hypoxemia at 16 weeks after CD19 CAR-T cell therapy. There was no evidence of CRS and any infections. The patient recovered after intravenous immunoglobulin without tocilizumab or glucocorticoid administration. Now he is still in remission without interstitial pneumonitis 3 years after CAR-T cell therapy.

**Conclusion:**

This is the first report of immunotherapy-associated interstitial pneumonitis after CAR-T cell therapy. Our finding suggested the importance of careful follow-up and proper treatments for immunotherapy-associated pneumonitis in the CAR-T cell therapy schedule.

## Introduction

Refractory or relapsed diffuse large B-cell lymphoma (DLBCL) patients have achieved nearly 50% complete response rate after receiving chimeric antigen receptor-modified T cells (CAR-T) targeting CD19 (1, 2). The most common adverse events reported are cytokine-release syndrome (CRS), and immune effector cell–associated neurotoxicity syndrome (ICANS) because of the overwhelming release of cytokines (3, 4), endothelial activation or increased blood–brain barrier permeability (5, 6). Due to the better understanding of these complications, at present there are mature grading systems and recommendations regarding toxicity management to ensure the safety of patients receiving CAR-T cell therapies (7, 8).The prompt administration of tocilizumab, a monoclonal antibody for IL-6 receptor, or glucocorticoids, has been served as the first-line therapy for CRS or ICANS (8).

Despite significant advancements in survival, CAR-T cell therapies are associated with unique and life-threatening complications. Besides high-grade CRS and ICANS, fatal hemophagocytic lymphohistiocytosis (HLH), macrophage activation syndrome (MAS) and lethal brain edema were reported (9). Pneumonitis caused by immune checkpoint inhibitors (ICIs) related adverse events (irAEs) has been reported with an incidence of 2.7-20% (10). But so far, there has been no report of immunotherapy-associated interstitial pneumonitis caused by CAR-T cell therapy. Here we report a 63-year-old male patient with refractory EBV-positive DLBCL, who developed immunotherapy-associated interstitial pneumonitis with prolonged hypoxemia after CD19 CAR-T cell therapy. With the accumulation of clinical data and increasing reports, we can better identify these complications and provide proper treatments.

## Case Report

**Figure 1:**
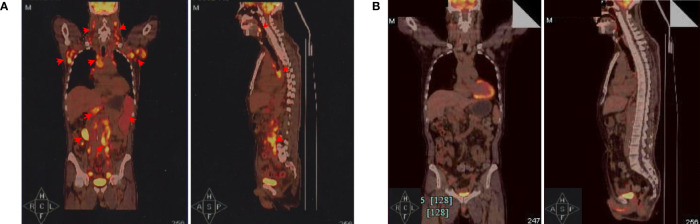
Positron emission tomography and computed tomography (PET-CT) evaluations. **(A)** PET-CT before CAR-T cell infusion. The coronal image (left) and sagittal image are shown (right). Red arrows indicate lymphoma lesions. **(B)** PET-CT examination showed significantly diminished lymphoma lesions and proved complete metabolic remission (CMR) 3 months after CAR-T cell therapy.

**Figure 2:**
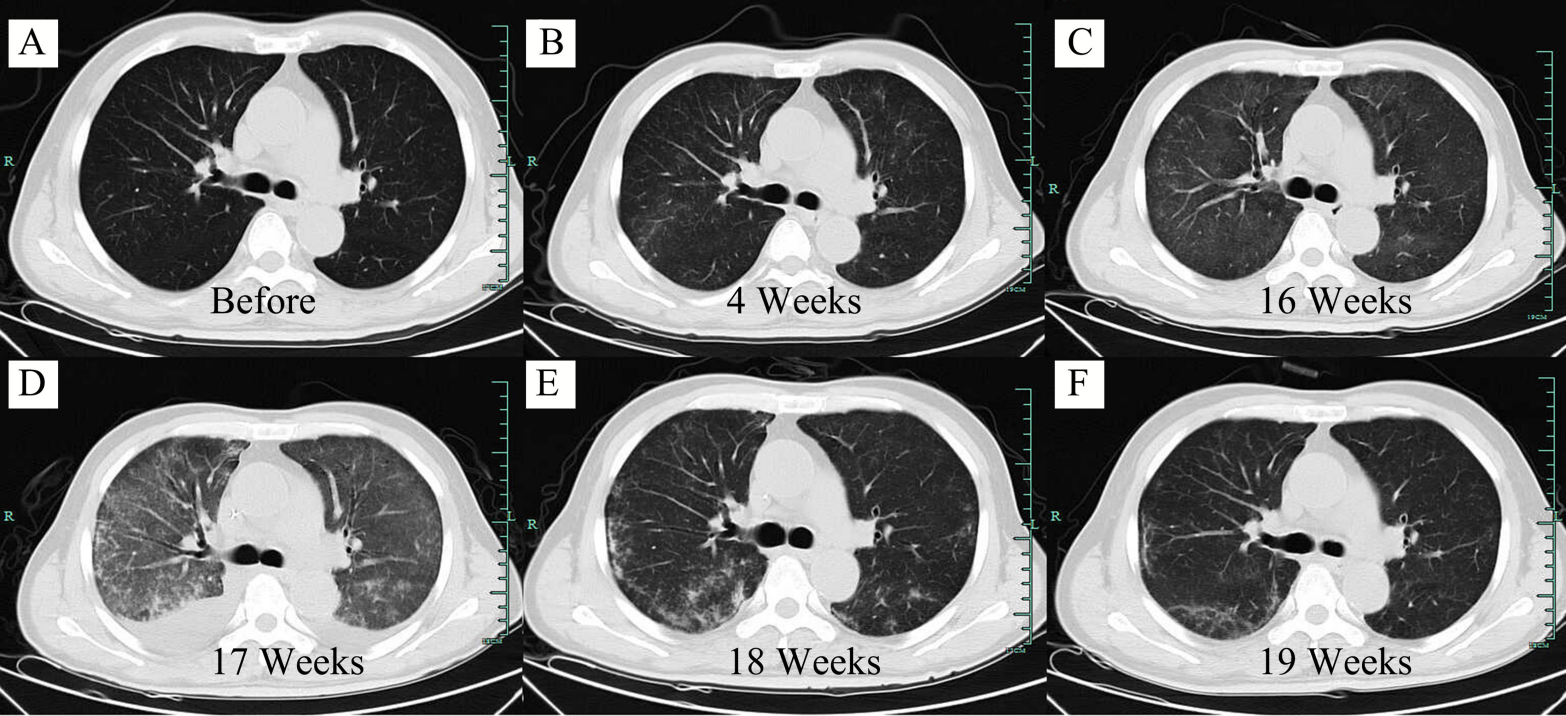
Axial computed tomography (CT) scan of the lung at different time points. **(A)** Screening period before CAR-T cell infusion. **(B)** 4 weeks after CAR-T cell infusion. **(C)** 16 weeks after CAR-T cell infusion. **(D)** 17 weeks after CAR-T cell infusion. **(E)** 18 weeks after CAR-T cell infusion. **(F)** 19 weeks after CAR-T cell infusion.

A 63-year-old Chinese male with bilateral cervical lymphadenopathy was diagnosed with EBV-positive DLBCL through a right cervical lymph node biopsy 40 months ago. Immunohistochemistry analysis showed CD3 (–), CD19 (+), CD20 (+), CD30 (+), CD5 (–), CD10 (-), BCL2 (+), BCL6 (-), MUM1 (+), PAX5 (+), C-MYC (-), ALK (-) and Ki-67 (+, 80%). *In situ* hybridization for EBV-encoded small RNA (EBER) was confirmed to be positive and EBV DNA was detectable in blood serum with a level of 5.44×10^6^ copies/ml. The patient underwent 7 courses of chemotherapies without any response, including 2 courses of R-CHOP, one course of R-GDP, 3 courses of R2-MINE, and one course of R2-DHAP. Then the patient was referred to our center and was recruited into a clinical trial of CD19 CAR-T cell therapy with a costimulatory 4-1BB endodomain (ClinicalTrials.gov ID: NCT02644655). The positron emission tomography and computed tomography (PET-CT) before CAR-T cell therapy revealed multiple lymphoma lesions with increased 18F-FDG metabolism (**Figure 1A**). CT scan showed no obvious pulmonary infection before CAR-T cell therapy (**Figure 2A**).

The patient received lymphodepleting chemotherapy with fludarabine (30 mg/m^2^) and cyclophosphamide (500 mg/m^2^) for 3 days (day −5 to −3). Subsequently, a total of 1.26×10^8^ CD19 CAR-T cells (2×10^6^ cells per kilogram) was infused on day 0. During the CAR-T cell therapy, the patient received sulfamethoxazole tablets (SMZ) for infection prevention. After CAR-T cell infusion, the patient developed grade 1 CRS and grade 4 neutropenia with fever 3 days later. According to the American society for transplantation and cellular therapy guidelines, piperacillin tazobactam and fluconazole were used in combination as anti-infective therapy with supportive care. One week later, all these symptoms resolved. PET-CT examination 3 months after CAR-T cell therapy showed regression of all lesions and complete metabolic remission was achieved (**Figure 1B**). Notably, serum EBV DNA was also undetectable.

**Figure 3:**
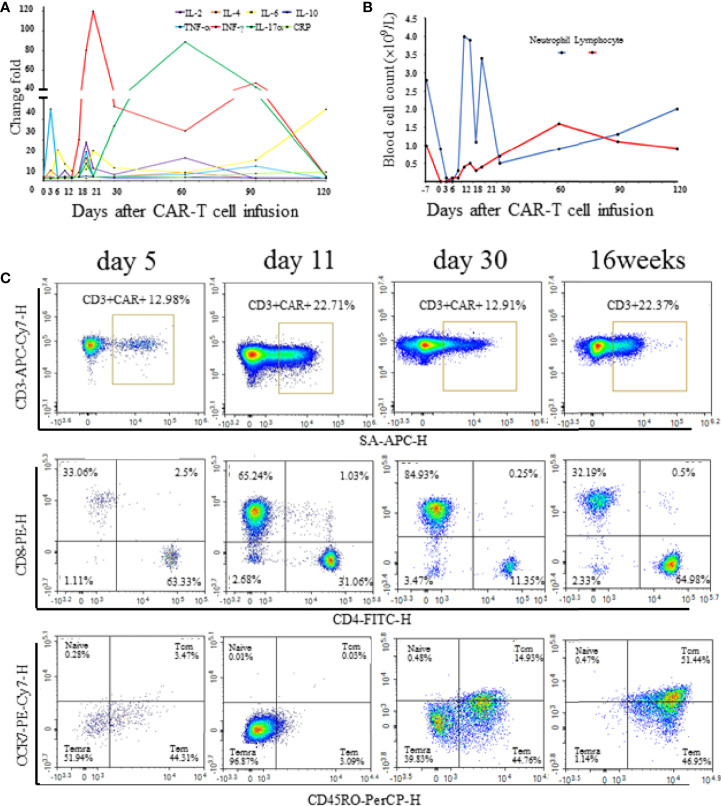
**(A)** The serum cytokine and C-reactive protein levels at different time points after CAR-T cell infusion. Change folds are relative to the values of day -7 before chemotherapy. **(B)** Neutrophil and lymphocyte count at different time points after CAR-T cell infusion. **(C)** Proportion and subtype of CAR-T cell at different time points after CAR-T cell infusion.

However, at 4 weeks after CAR-T cell therapy, the CT scan showed mild bilateral lung diffuse lesions without any symptoms or signs (**Figure 2B**), so no treatment was given. 16 weeks after CAR-T cell therapy, the patient developed prolonged hypoxemia without fever. The CT scan confirmed interstitial pneumonitis with a small amount of pleural effusion (**Figure 2C**), which progressed one week later (**Figure 2D**). Influenza virus, COVID-19 and cytomegalovirus infection were excluded through epidemiologic features and laboratory tests. Bronchoscopy and bronchoalveolar lavage fluid examination also failed to capture any abnormalities. PCR examination confirmed the existence of CAR construct DNA copies in peripheral blood. IFN-γ and IL-17a level was increased before the development of pneumonitis, while the IL-6 level and lymphocyte count were significantly increased at the diagnosis of the pneumonitis (**Figures 3A, B**). The proportion of CAR-T cells re-amplificated, the subtype of CAR-T cells turned from CD8+ T cells back to CD4+ T cells, and the ratio of central memory T cells increased continually at the diagnosis of the pneumonitis (**Figure 3C**). Given the time onset and all evidences, CRS was excluded and the diagnosis of immunotherapy-associated interstitial pneumonitis after CAR-T cell therapy was made. Therefore, no tocilizumab was given. To avoid any potential negative effects on CAR-T cells, glucocorticoid administration was not given, although this was considered the preferred treatment for interstitial pneumonitis. Finally, intravenous immunoglobulin of 10 grams daily with intravenous levofloxacin was given with careful supportive treatment. Fortunately, the patient recovered from dyspnea 1 week later and the CT scan showed absorption of lesions (**Figures 2E, F**). At present, the patient is still in remission without interstitial pneumonitis 3 years after CAR-T cell therapy.

This study was approved by the Human Ethics Committee of the Second Affiliated Hospital, School of Medicine, Zhejiang University, China (the number of approvals: SAHZ-2018–036). Written informed consent was obtained from the patient’s family for publication of this case report and any accompanying images.

## Discussions

Over the last few years, immunotherapy experienced rapid development with more and more promising immunotherapeutic modalities (11). Currently, tumor immunotherapies mainly refer to CAR-T cell therapy and immune checkpoint inhibitors (ICIs), the latter including monoclonal antibodies targeting programmed cell death protein 1 (PD-1), programmed death-ligand 1 (PD-L1) and cytotoxic T lymphocyte-associated antigen-4 (CTLA-4). The investigations about these ICIs in malignant lymphoma have generated promising results in preclinical studies and/or clinical trials (12). Pneumonitis caused by ICIs has been studied and reported in detail. Immune-related pneumonitis can be severe or even potentially life-threatening. The onset time of this complication was approximately 3 months after the first dose of ICIs (13, 14). Currently, the mechanisms for immune-related pneumonitis are poorly understood. Increased inflammatory cytokines are one of the possible mechanisms (15). IL-6 seems to play an essential role in immune-related pneumonitis, and Th17 cells enhanced and polarized through IL-6 expression, may serve as a more precise biomarker (16). IL-17 plays a crucial role in mediating the pro-inflammatory immune response and elevated IL-17 predisposes to the development of pneumonitis (17). IFN-γ levels were decreased compared with baseline in patients who developed immune-related pneumonitis (18). Here we reported the first documented case of immunotherapy-associated interstitial pneumonitis at 16 weeks after the CAR-T cell therapy, when it was not in the peak period of CRS. In our case, IFN-γ and IL-17a were increased before the development of pneumonitis, whereas IL-6 was elevated at the diagnosis of pneumonitis. These chemokines are biomarkers for immune-related pneumonitis prediction and diagnosis (19).

CD19 CAR-T-cell therapy has emerged as a promising therapy for patients with B-cell malignancies. Three anti-CD19 CAR-T cell constructs with different costimulatory ednodomain are already clinically available. In CAR T-cell therapy recipients, pulmonary complications are more frequent in patients with higher-grade CRS. The most frequent pulmonary symptom was hypoxia, pleural effusion, pulmonary embolism, allergic rhinitis, and pneumomediastinum (20). Most of these pulmonary complications were attributed to the manifestations of CRS and occurred within 10 days after CAR T-cell infusion (20). Due to greater degrees of immune impairment following CAR-T therapy, pneumonia is common and may be life-threatening. The challenge of CAR-T cell therapy is in distinguishing between infection and CRS. In one series, 80% of first infections were within the first 10 days after CAR-T infusion. Late-onset infections were rare and may reflect prolonged immunoglobulin deficiency (21). Bronchoscopy and bronchoalveolar lavage fluid examination were negative for our patient, ruling out infectious etiologies and tumor progression. Given the time onset and all evidences, CRS and pneumonia were excluded, and the diagnosis of immunotherapy-associated interstitial pneumonitis after CAR-T cell therapy was made.

Several guidelines have been established for grading the severity of interstitial pneumonitis and guiding the proper treatments (22, 23). At present, the agents usually used for interstitial pneumonitis caused by irAEs include: (1) glucocorticoids; (2) infliximab which inhibits inflammation by blocking TNF-α binding to its receptor; (3) mycophenolate mofetil which inhibits the function of T and B cells; (4) cyclophosphamide; (5) intravenous immunoglobulin which can serve an immune-regulatory role to inhibit inflammation through complement pathway, humoral immunity and cellular immunity. According to the irAEs guidelines from the European society for medical oncology and society for immunotherapy of cancer, our patient suffered from grade 3 interstitial pneumonitis, which needs medium to large doses of intravenous glucocorticoids or even immunosuppressive agents. To avoid any potential negative effects on CAR-T cells, our patient did not receive any glucocorticoid administration, not to mention other immunosuppressive agents. But we believe the appropriate glucocorticoids administration is necessary for more severe potential patients in the future.

While CRS and ICANS are the most common CAR-T cell therapy toxicities, the case we presented here highlighted the importance of mounting potential immune-related pulmonary pneumonitis after the CAR-T cell therapy. Early detection and diagnosis, and appropriate management according to the severity are critical to improving the prognosis. We hope that our experience will inform patients and physicians alike about the rare immunotherapy-adverse effects of CAR-T cell therapy.

## Data Availability Statement

The original contributions presented in the study are included in the article/supplementary material. Further inquiries can be directed to the corresponding authors.

## Ethics Statement

The studies involving human participants were reviewed and approved by Human Ethics Committee of the Second Affiliated Hospital, School of Medicine, Zhejiang University, China (the number of approvals: SAHZ-2018–036). The patients/participants provided their written informed consent to participate in this study.

## Author Contributions

Conceptualization: XY and HL. Data curation: ZS and CX. Formal analysis: ZS. Funding acquisition: HL. Resources: XY and HL. Supervision: XY and HL. Validation: ZS and CX. Writing – original draft: ZS. Writing – review & editing: XY and HL. All authors contributed to the article and approved the submitted version.

## Funding

This work was financially supported by the Zhejiang Natural Science Foundation (No. LY18H160008).

## Conflict of Interest

The authors declare that the research was conducted in the absence of any commercial or financial relationships that could be construed as a potential conflict of interest.

## Publisher’s Note

All claims expressed in this article are solely those of the authors and do not necessarily represent those of their affiliated organizations, or those of the publisher, the editors and the reviewers. Any product that may be evaluated in this article, or claim that may be made by its manufacturer, is not guaranteed or endorsed by the publisher.

## Abbreviations

DLBCL, large B-cell lymphoma; CAR-T, chimeric antigen receptor-modified T cells; CRS, cytokine-release syndrome; ICANS, immune effector cell–associated neurotoxicity syndrome; ICIs, immune checkpoint inhibitors; irAEs, immune checkpoint inhibitors (ICIs) related adverse events; EBER, EBV-encoded small RNA; PET-CT, positron emission tomography and computed tomography.

## References

[1] Schuster SJ Bishop MR Tam CS Waller EK Borchmann P McGuirk JP. Tisagenlecleucel in Adult Relapsed or Refractory Diffuse Large B-Cell Lymphoma. N Engl J Med (2019) 380(1):45–56. doi: 10.1056/NEJMoa1804980 30501490

[2] Neelapu SS Locke FL Bartlett NL Lekakis LJ Miklos DB Jacobson CA. Axicabtagene Ciloleucel CAR T-Cell Therapy in Refractory Large B-Cell Lymphoma. N Engl J Med (2017) 377(26):2531–44. doi: 10.1056/NEJMoa1707447 PMC5882485 29226797

[3] Giavridis T van der Stegen SJC Eyquem J Hamieh M Piersigilli A Sadelain M. CAR T Cell-Induced Cytokine Release Syndrome Is Mediated by Macrophages and Abated by IL-1 Blockade. Nat Med (2018) 24(6):731–8. doi: 10.1038/s41591-018-0041-7 PMC6410714 29808005

[4] Norelli M Camisa B Barbiera G Falcone L Purevdorj A Genua M. Monocyte-Derived IL-1 and IL-6 Are Differentially Required for Cytokine-Release Syndrome and Neurotoxicity Due to CAR T Cells. Nat Med (2018) 24(6):739–48. doi: 10.1038/s41591-018-0036-4 29808007

[5] Santomasso BD Park JH Salloum D Riviere I Flynn J Mead E. Clinical and Biological Correlates of Neurotoxicity Associated With CAR T-Cell Therapy in Patients With B-Cell Acute Lymphoblastic Leukemia. Cancer Discov (2018) 8(8):958–71. doi: 10.1158/2159-8290.CD-17-1319 PMC6385599 29880584

[6] Gust J Hay KA Hanafi L-A Li D Myerson D Gonzalez-Cuyar LF. Endothelial Activation and Blood-Brain Barrier Disruption in Neurotoxicity After Adoptive Immunotherapy With CD19 CAR-T Cells. Cancer Discov (2017) 7(12):1404–19. doi: 10.1158/2159-8290.CD-17-0698 PMC5718945 29025771

[7] Lee DW Gardner R Porter DL Louis CU Ahmed N Jensen M. Current Concepts in the Diagnosis and Management of Cytokine Release Syndrome. Blood (2014) 124(2):188–95. doi: 10.1182/blood-2014-05-552729 PMC4093680 24876563

[8] Lee DW Santomasso BD Locke FL Ghobadi A Turtle CJ Brudno JN. ASTCT Consensus Grading for Cytokine Release Syndrome and Neurologic Toxicity Associated With Immune Effector Cells. Biol Blood Marrow Transplant (2019) 25(4):625–38. doi: 10.1016/j.bbmt.2018.12.758 PMC12180426 30592986

[9] Neelapu SS Tummala S Kebriaei P Wierda W Gutierrez C Locke FL. Chimeric Antigen Receptor T-Cell Therapy - Assessment and Management of Toxicities. Nat Rev Clin Oncol (2018) 15(1):47–62. doi: 10.1038/nrclinonc.2017.148 28925994 PMC6733403

[10] Nishino M Giobbie-Hurder A Hatabu H Ramaiya NH Hodi FS. Incidence of Programmed Cell Death 1 Inhibitor-Related Pneumonitis in Patients With Advanced Cancer: A Systematic Review and Meta-Analysis. JAMA Oncol (2016) 2(12):1607–16. doi: 10.1001/jamaoncol.2016.2453 27540850

[11] Mihalyova J Hradska K Jelinek T Motais B Celichowski P Hajek R. Promising Immunotherapeutic Modalities for B-Cell Lymphoproliferative Disorders. Int J Mol Sci (2021) 22(21):11470. doi: 10.3390/ijms222111470. 34768899 PMC8584080

[12] Lin N Song Y Zhu J. Immune Checkpoint Inhibitors in Malignant Lymphoma: Advances and Perspectives. Chin J Cancer Res (2020) 32(3):303–18. doi: 10.21147/j.issn.1000-9604.2020.03.03 PMC7369179 32694896

[13] Naidoo J Wang X Woo KM Iyriboz T Halpenny D Cunningham J. Pneumonitis in Patients Treated With Anti-Programmed Death-1/Programmed Death Ligand 1 Therapy. J Clin Oncol (2017) 35(7):709–17. doi: 10.1200/JCO.2016.68.2005 PMC5559901 27646942

[14] Delaunay M Cadranel J Lusque A Meyer N Gounant V Moro-Sibilot D. Immune-Checkpoint Inhibitors Associated With Interstitial Lung Disease in Cancer Patients. Eur Respir J (2017) 50(2):1700050. doi: 10.1183/13993003.00050-2017 28798088

[15] Postow MA Sidlow R Hellmann MD. Immune-Related Adverse Events Associated With Immune Checkpoint Blockade. N Engl J Med (2018) 378(2):158–68. doi: 10.1056/NEJMra1703481 29320654

[16] Kowalski B Valaperti A Bezel P Steiner UC Scholtze D Wieser S. Analysis of Cytokines in Serum and Bronchoalveolar Lavage Fluid in Patients With Immune-Checkpoint Inhibitor-Associated Pneumonitis: A Cross-Sectional Case-Control Study. J Cancer Res Clin (2021). doi: 10.1007/s00432-021-03750-z PMC9189083 34347128

[17] Tyan K Baginska J Brainard M Giobbie-Hurder A Severgnini M Manos M. Cytokine Changes During Immune-Related Adverse Events and Corticosteroid Treatment in Melanoma Patients Receiving Immune Checkpoint Inhibitors. Cancer Immunol Immunother (2021) 70(8):2209–21. doi: 10.1007/s00262-021-02855-1 PMC10991353 33481042

[18] Hirashima T Kanai T Suzuki H Yoshida H Matsushita A Kawasumi H. The Levels of Interferon-Gamma Release as a Biomarker for Non-Small-Cell Lung Cancer Patients Receiving Immune Checkpoint Inhibitors. Anticancer Res (2019) 39(11):6231–40. doi: 10.21873/anticanres.13832 31704852

[19] von Itzstein MS Khan S Gerber DE. Investigational Biomarkers for Checkpoint Inhibitor Immune-Related Adverse Event Prediction and Diagnosis. Clin Chem (2020) 66(6):779–93. doi: 10.1093/clinchem/hvaa081 PMC7259479 32363387

[20] Wudhikarn K Pennisi M Garcia-Recio M Flynn JR Afuye A Silverberg ML. DLBCL Patients Treated With CD19 CAR T Cells Experience a High Burden of Organ Toxicities But Low Nonrelapse Mortality. Blood Adv (2020) 4(13):3024–33. doi: 10.1182/bloodadvances.2020001972 PMC7362382 32614964

[21] Hill JA Li D Hay KA Green ML Cherian S Chen X. Infectious Complications of CD19-Targeted Chimeric Antigen Receptor-Modified T-Cell Immunotherapy. Blood (2018) 131(1):121–30. doi: 10.1182/blood-2017-07-793760 PMC5755046 29038338

[22] Puzanov I Diab A Abdallah K Bingham CO Brogdon C Dadu R. Managing Toxicities Associated With Immune Checkpoint Inhibitors: Consensus Recommendations From the Society for Immunotherapy of Cancer (SITC) Toxicity Management Working Group. J Immunother Cancer (2017) 5(1):95. doi: 10.1186/s40425-017-0300-z 29162153 PMC5697162

[23] Haanen JBAG Carbonnel F Robert C Kerr KM Peters S Larkin J. Management of Toxicities From Immunotherapy: ESMO Clinical Practice Guidelines for Diagnosis, Treatment and Follow-Up. Ann Oncol (2017) 28(suppl_4):iv119–42. doi: 10.1093/annonc/mdx225 28881921

